# Therapeutic and Diagnostic Potential of a Novel K1 Capsule Dependent Phage, JSSK01, and Its Depolymerase in Multidrug-Resistant *Escherichia coli* Infections

**DOI:** 10.3390/ijms252312497

**Published:** 2024-11-21

**Authors:** Naveen Gattuboyena, Yu-Chuan Tsai, Ling-Chun Lin

**Affiliations:** 1 Master Program in Biomedical Sciences, School of Medicine, Tzu Chi University, No. 701, Sec. 3, Zhongyang Road, Hualien 97004, Taiwan; 109329105@gms.tcu.edu.tw; 2 Institute of Medical Sciences, Tzu Chi University, No. 701, Sec. 3, Zhongyang Road, Hualien 97004, Taiwan; 111353101@gms.tcu.edu.tw

**Keywords:** multidrug resistance, K1 capsule, phage therapy, endo-N-acetylneuraminidase, *Escherichia coli*, depolymerase

## Abstract

Bacteriophages are viruses that have the potential to combat bacterial infections caused by antimicrobial-resistant bacterial strains. In this study, we investigated a novel lytic bacteriophage, vB_EcoS_JSSK01, isolated from sewage in Hualien, Taiwan, which effectively combats multidrug-resistant (MDR) *Escherichia coli* of the K1 capsular type. K1 *E. coli* is a major cause of severe extraintestinal infections, such as neonatal meningitis and urinary tract infections. Phage JSSK01 was found to have a genome size of 44,509 base pairs, producing approximately 123 particles per infected cell in 35 min, and was highly stable across a range of temperatures and pH. JSSK01 infected 59.3% of the MDR strains tested, and its depolymerase (ORF40) specifically degraded the K1 capsule in these bacteria. In a zebrafish model, JSSK01 treatment after infection significantly improved survival, with survival in the treated group reaching 100%, while that in the untreated group dropped to 10% after three days. The functional activity of depolymerase was validated using zone inhibition and agglutination tests. These results indicate that JSSK01 and its substrate-specific depolymerase have promising therapeutic and diagnostic applications against K1-encapsulated MDR *E. coli* infections.

## 1. Introduction

*Escherichia coli* is a gram-negative bacterium commonly found in the gastrointestinal tract of animals, where it often establishes a beneficial symbiotic relationship with its host [1,2]. However, certain *E. coli* variants have evolved pathogenic traits that allow them to mimic host receptors, allowing them to evade the immune system and cause severe infections [1,3,4,5,6]. These pathogenic strains can colonize both gastrointestinal and extraintestinal sites, leading to various conditions, such as cystitis, pyelonephritis, cellulitis, pneumonia, and postoperative infections [1,7]. One significant group of these pathogenic strains is extraintestinal *E. coli* (ExPEC), which are part of the gut microbiota but harbor virulence factors, enabling them to colonize various sites and exhibit increased antibiotic resistance [8,9,10,11,12]. As a result, *E. coli* of the K1 capsular polysaccharide (CPS) can cause infections, such as neonatal meningitis, urinary tract infections, and bloodstream infections [5,7,13], and the frequency of these infections is increasing.

The structure of the K1 capsule is similar to that of polysialic acid found in the mammalian neural cell adhesion molecule [3], and this similarity allows *E. coli* to evade phagocytosis by immune cells [14]. This promotes survival beyond the gut and bloodstream and crossing of the blood-brain barrier, increasing the risk of infections, such as neonatal meningitis, late-onset sepsis, and early-onset sepsis [14,15,16,17]. The World Health Organization recommends using antibiotics, such as penicillin (ampicillin), aminoglycosides (gentamicin), or third-generation cephalosporins (such as ceftriaxone), to treat bacterial meningitis [18,19]. However, ampicillin resistance is common, and studies have identified extended-spectrum beta-lactamase and multidrug-resistant (MDR) *E. coli* strains that complicate treatment [18,20,21]. Moreover, the ongoing antimicrobial resistance (AMR) epidemic renders many antibiotics ineffective against these infections. Further, the combination of pathophenotypes (disease-causing characteristics) and AMR poses significant challenges for treatment. Therefore, alternative approaches that can simultaneously target both the pathophenotypes and AMR of K1 *E. coli* are urgently needed.

Bacteriophages, commonly referred to as phages, are viruses that specifically infect bacteria. Bacteriophages offer an attractive platform for treating AMR bacterial infections [22] due to their capability to infect bacteria and utilize the host’s replication machinery to synthesize new progeny within the bacterial cytoplasm. Upon release, new phage particles destroy the bacterial cell membrane, ultimately leading to bacterial cell death. Notably, the antibiotic resistance mechanisms employed by bacteria do not interfere with the phage’s ability to recognize and infect its host, allowing highly specific targeting of bacterial infections via phages [23,24]. In recent years, phage therapy has shown remarkable promise in the treatment of MDR bacterial infections by successfully addressing challenges posed by bacteria resistant to multiple antibiotics and providing hope for treating infections that were previously difficult to manage with conventional antibiotics [24,25,26]. Phages can be utilized through their replication kinetics for therapy and by using their unique protein machineries as antimicrobials, such as endolysins and depolymerases [27]. In particular, phage-derived depolymerases have garnered attention in recent years for their potential applications in various fields, including phage therapy and biofilm disruption for combating bacterial infections [27,28,29,30,31,32], as their ability to degrade bacterial protective structures makes them valuable tools for researchers and medical professionals working to combat antibiotic-resistant bacteria and improve treatment modalities. Owing to their high target specificity, these depolymerases also provide a promising method for bacterial typing [28,33,34].

Research on K1 *E. coli* phages has been limited despite their potential to mitigate AMR bacterial infections. Moreover, the increasing prevalence of antibiotic resistance makes it critical to pursue further research on bacteriophages. In this study, we aimed to isolate and characterize a novel bacteriophage from environmental sewage water from hospitals. The isolated phage, designated JSSK01, belonged to the siphophage family and specifically infected *E. coli* strains possessing K1 CPS. We conducted a detailed characterization of JSSK01 through comprehensive sequence analysis and comparison with previously identified phages. Notably, JSSK01 encoded a depolymerase domain within its tail spike. We expressed this tail spike protein and confirmed its specificity for degrading the K1 capsule. Therefore, besides using bacteriophages in phage therapy, the tail spike protein, due to its specificity for K1 CPS, can also shorten the time required for *E. coli* K1 type identification.

## 2. Results

### 2.1. Antibiotic Susceptibility of Phage-Sensitive Bacterial Strains

Four *E. coli* strains (Table 1) were clinical isolates obtained from Buddhist Tzu Chi Hospital, Hualien, Taiwan, and selected for antibiotic susceptibility testing. Two of these strains 70751 (the host of JSSK01) and 78030 were characterized as MDR, showing resistance to up to five different classes of antibiotics. In contrast, G0032 and G0218 displayed resistance to at least one class of antibiotics and intermediate resistance to another class.

### 2.2. Morphology of JSSK01 Under Transmission Electron Microscopy (TEM)

**Figure 1:**
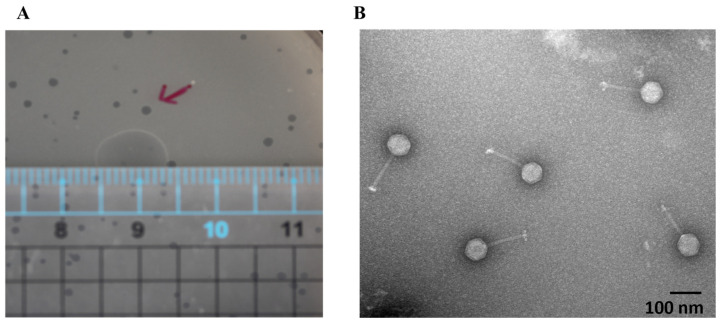
JSSK01 morphology under transmission electron microscopy. (**A**) JSSK01 plaque formation in a plaque assay measuring 0.1–0.15 cm; the red arrows represent typical plaques. (**B**) JSSK01 phage is classified as a siphophage with a 65 ± 4 nm (n = 10) icosahedral head and a 124 ± 6 nm (n = 10) nm long tail, electron micrograph of JSSK01 virions with scale bar of 100 nm, viewed at 150,000 × magnification under negative staining with 2% uranyl acetate.

A novel phage was isolated from sewage water in eastern Taiwan via a mixed culture of *E. coli* strains. One showed a positive response, forming clear plaques on bacterial lawns (Figure 1A). After CsCl purification, TEM revealed crucial morphological characteristics (Figure 1B). The phage had an icosahedral head measuring 65 ± 4 nm (n = 10) and a long tail measuring 124 ± 6 nm (n = 10). These features classify the isolated phage as a member of the siphophage group. The systematic name assigned to this phage is vB_EcoS_JSSK01 (JSSK01).

### 2.3. Biological Properties and Lytic Ability of JSSK01

**Figure 2:**
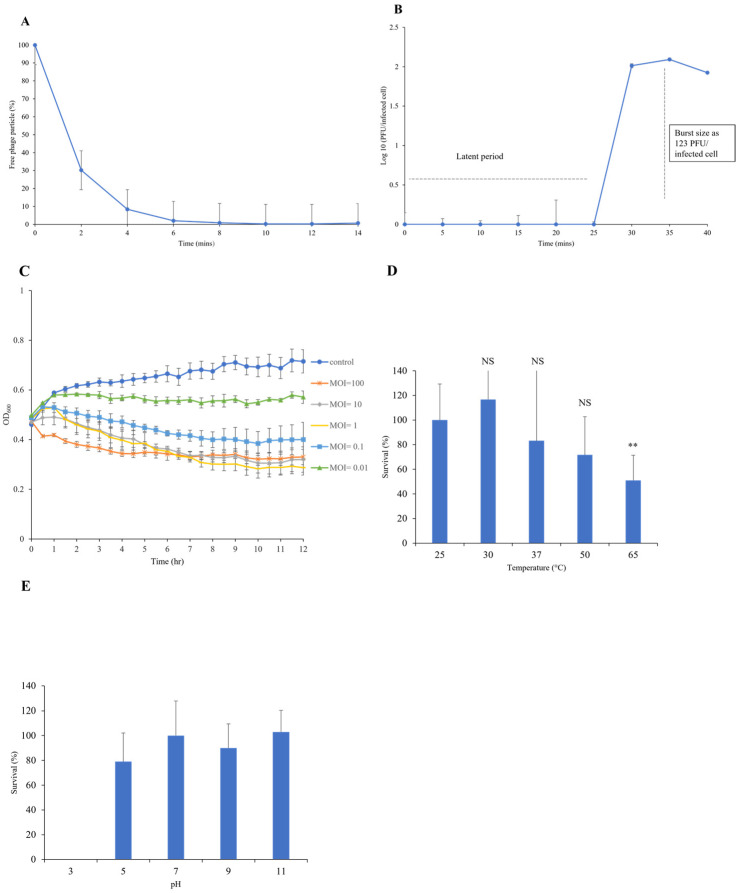
Biological properties, lytic activity, and stability of JSSK01. (**A**) Approximately 95% of the phage was adsorbed onto the host multidrug-resistant (MDR) *E. coli* 70751 within 6 min and was completely adsorbed by 10 min. (**B**) One-step growth curve of JSSK01 on MDR 70751. The phage undergoes a 25-min latent period, releasing approximately 123 phage-forming units (PFUs) per infected cell at 35 min. (**C**) JSSK01 lytic activity on MDR 70751. A 0.5 OD_600_ of MDR 70751 was used to infect the phage at different MOIs and monitored for 12 h by measuring the OD_600_ every 30 min. (**D**) JSSK01 stability was assessed by exposing it to different temperatures, ranging from 25 °C to 65 °C, for 1 h. ** indicate*s p* < 0.01; NS denotes no significance. (**E**) JSSK01 stability was assessed by incubating 10^8^ PFU/mL phage titers in pH-adjusted LB media for 1 h. Plaque assays were performed to determine phage survival after treatment. All the experiments were performed in triplicate; the error bars represent the standard error of the mean.

A series of assays were conducted to characterize the growth kinetics and lytic ability of the JSSK01 phage by infecting the host bacterial strain MDR70751 at various multiplicities of infection (MOIs). The JSSK01 phage demonstrated complete adsorption to its host strain 70751 after 10 min (Figure 2A). The life cycle of JSSK01 involves a latent phase of 25 min followed by a steady release of new phage particles at 35-min intervals. On average, a single infected bacterial cell produced ~123 phage particles (Figure 2B). The bacteriolytic activity was measured by monitoring the optical density at 600 nm (OD_600_). As shown in Figure 2C, when the host strain 70751 was infected at different MOIs (0.01, 0.1, 1, 10, and 100), the phage effectively inhibited bacterial growth for 5 h without phage emergence. However, higher MOIs (1, 10, and 100) were the most effective at rapidly reducing bacterial growth. Low MOIs (0.01 and 0.1) also inhibited bacterial growth in a time-dependent manner.

### 2.4. Thermal and pH Stability of JSSK01

The stability of JSSK01 was evaluated by exposing it to various temperatures and pH values. As shown in Figure 2D, the phage retained full activity at temperatures of 25, 30, and 37 °C. However, phage titers were reduced at 50 °C by 70% and significantly decreased by 40% at 65 °C. As shown in Figure 2E, a pH test was conducted by exposure to alkaline and acidic conditions. The phage completely lost infectivity at pH 3. However, at pH 5, the phage titers decreased by 20% and remained infective. The phages exhibited optimal stability at pH 7, 9, and 11, with minimal or no effect on infectivity. These results suggest that the stability of JSSK01 is compromised under extreme conditions, such as high temperature (65 °C) and strong acidity (pH 3).

### 2.5. General Genomic Properties and Structural Proteins of JSSK01

**Figure 3:**
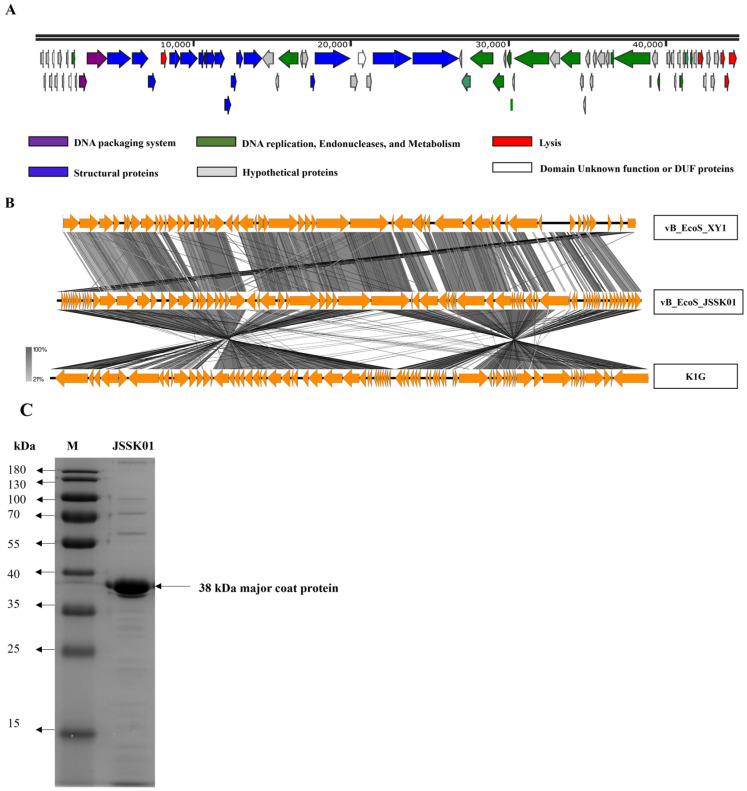
JSSK01 genome organization, comparison, and structural protein analysis. (**A**) Each arrow represents an open reading frame (ORF). Based on their encoded protein function, a color was assigned to each ORF. (**B**) The JSSK01 phage genome was compared via Easyfig with those of two other similar phages, *Escherichia* siphophages XY1 and K1G. (**C**) The protein structure of JSSK01 was analyzed via sodium dodecyl sulfate-polyacrylamide gel electrophoresis. The gel was visualized via an iBright imaging system, and the major coat protein was identified at 38 kDa and confirmed via tandem mass spectrometry.

The complete genome sequence of JSSK01 was 44,509 bp in size, with 50.0% GC content. No terminal repeats or known tRNA genes were found. The genome sequence contained 80 predicted open-reading frames (ORFs), with 54 located on the positive DNA strand and 26 on the negative strand. The putative functions of these ORFs were predicted via BLASTp, PSI-BLAST, and conserved domain search tools. Approximately 38 ORFs were classified as hypothetical proteins, three of which had unknown functional domains. A total of 39 proteins with various functions, including in DNA replication, endonuclease activity, and metabolism, were annotated (Table S1 and Figure 3A). These proteins included a transcriptional regulator (ORF11), calcineurin-like phosphodiesterase (ORF31), DNA primase (ORF42), helicase (ORF43), DNA cytosine methyltransferase (ORF44), VRR-NUC domain protein (ORF46), DNA polymerase (ORF49), putative nuclease (ORF51), helix-turn-helix XRE-family-like protein (ORF63), glycosyl hydrolase (ORF68), valyl-tRNA synthetase (ORF70), and dextransucrase (ORF71). Structural proteins essential for phage assembly were also identified, including major and minor capsid proteins (ORF20 and ORF16), tail proteins (ORF17 and ORF39), head-tail connector proteins (ORF22, ORF23, ORF24, and ORF25), tail components, such as tube and completion proteins (ORF26, ORF27, ORF28, and ORF34), and a tail spike protein with endo-N-acetylneuraminidase (ENgase) activity (ORF40). Furthermore, proteins associated with the phage lysis system were identified, including putative spanin (ORF18), langerin-binding protein (ORF73), putative holin-like class II (ORF78), putative holin-like class I (ORF79), endolysin (ORF80), and two putative DNA packaging proteins known as terminase small and large subunits (ORF13 and ORF14). The genes described above constitute the complete set of genes for a lytic bacteriophage. Notably, no genes encoding toxins, integrases, or recombinases were found in the genome. These findings indicate that JSSK01 exhibits a lytic life cycle.

### 2.6. Genome Comparison Analysis

When comparing the JSSK01 genome with those of *Escherichia* phages vB_EcoS_XY1 (81% query coverage with 91% identity) and K1G (77% query coverage with 92.69% identity) (Figure 3B), notable similarities and differences were observed. JSSK01’s genome displayed high nucleotide homology with vB_EcoS_XY1, though 14 ORFs present in JSSK01 were absent in XY1. In contrast, K1G exhibited a larger discrepancy, missing 36 ORFs present in JSSK01. Many of these unique ORFs in JSSK01 encode hypothetical proteins, most with short polypeptides under 100 amino acids, potentially representing novel functionalities.

A smaller fraction of JSSK01’s ORFs could be functionally annotated; of its 80 ORFs, 39 were assigned specific roles. Key annotated ORFs not found in XY1 or K1G include ORF 44 (a DNA cytosine methyltransferase) [35], ORF 63 (a helix-turn-helix XRE-family transcriptional regulator), and ORF 68 (a glycosyl hydrolase). These proteins could confer unique benefits to JSSK01; for instance, the methyltransferase may play a role in host immune evasion, while the transcriptional regulator and glycosyl hydrolase [36,37] could influence gene expression and host cell wall degradation. Together, these differences may help explain JSSK01’s distinct adsorption and growth kinetics, highlighting its potential for applications where high host specificity and efficiency are desirable.

### 2.7. JSSK01 Coat Protein Identification

Structural protein analysis was performed via SDS–PAGE, which revealed approximately 17 protein bands (Figure 3C). Major protein bands were subjected to tandem mass spectrometry (MS/MS) analysis for identification. The analysis confirmed a putative major coat protein (ORF20) consisting of 349 amino acids with an estimated molecular weight of 38 kDa. This identification was consistent with the predictions from in silico analysis of the genome sequence.

### 2.8. Phylogenetic Characterization of JSSK01

**Figure 4:**
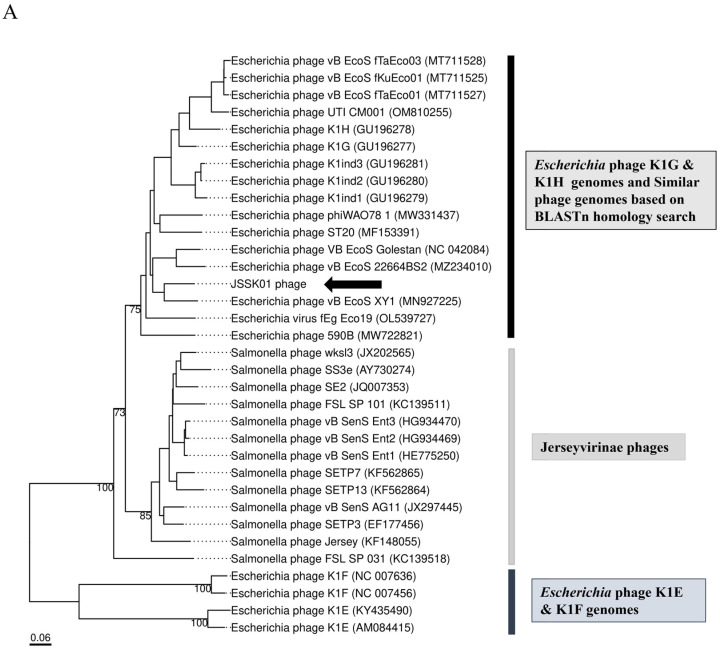

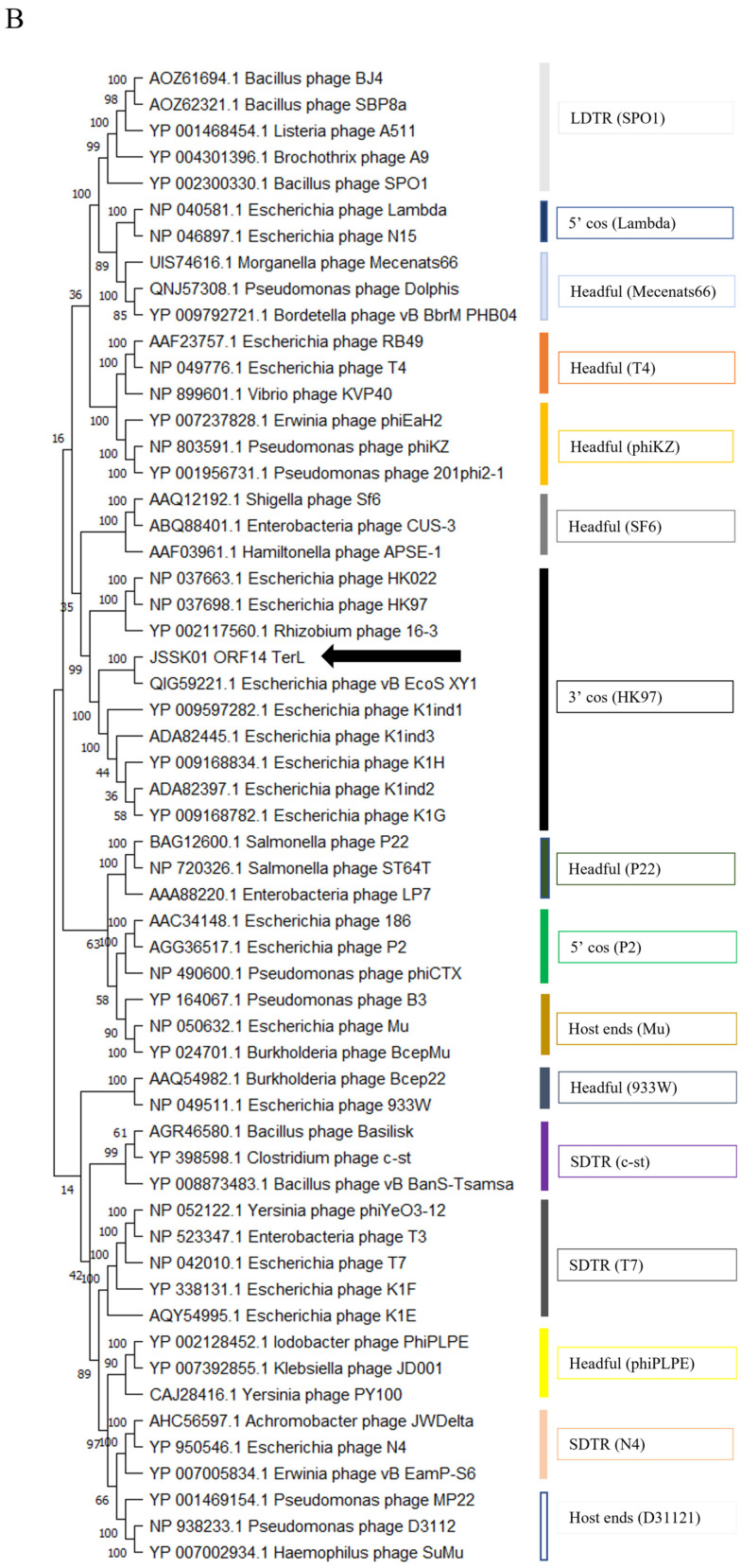

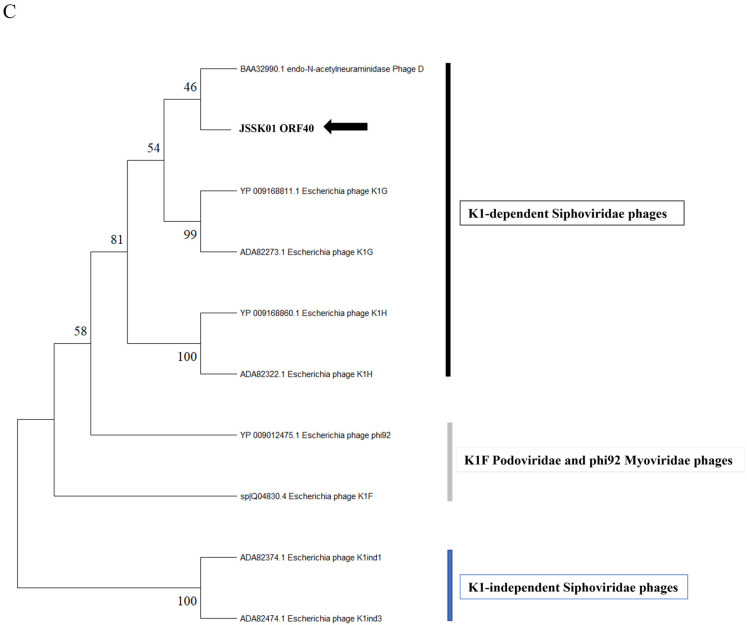
Phylogenetic analysis of JSSK01. (**A**) JSSK01 phylogenetic analysis via VICTOR. Phage genome analysis for genome comparison with K1 gikeviruses, Jerseylikeviruses, and K1 podophages. The genomic distance was inferred via the D6 formula (scale length of 0.06). The relatedness was 2 at the family and subfamily levels, 3 at the genus level, and 21 at the species level. (**B**) Terminase large subunit-based phylogenetic analysis was carried out via MEGA 11 software. The JSSK01 terminase (ORF14) was compared with K1 gikevirus terminases and classified under 3’cos (HK97) terminases. (**C**) JSSK01 endo-N-acetylneuraminidase (ENgase) or ORF40 phylogenetic analysis was performed with other tail spike proteins via MEGA 11. The amino acid sequences of all K1 *E. coli* strains recognized by phage tail spike proteins were collected, and phylogenetic analysis was performed via the neighbor-joining method. The ORF40 of JSSK01 was grouped with K1-dependent tail spike proteins. For all the trees, the position of JSSK01 is marked with a black arrow.

To trace the evolutionary relationships of JSSK01, a genome-based phylogenetic analysis was performed via VICTOR. The constructed proteomic tree consisted of siphophages belonging to the order Caudovirales, family *Siphoviridae*, and subfamily “*Jerseyvirinae*”. This subfamily included three genera: “*Jerseylikevirus*”, “*Sp3unalikevirus*”, and “*K1gikevirus*”, with 13 *Salmonella*-specific phages belonging to Jerseylikevirus, 16 *Escherichia*-specific K1G, K1H, and other similar phages, and 4 K1E and K1F podophages belonging to “*K1glikevirus*”. As shown in Figure 4A, the tree diverged into 2 main branches at the family and subfamily levels, 3 at the genus level, and 21 at the species level. The tree revealed that the JSSK01 phage belonged to the “*K1glikevirus*” genus. TerL is a conserved genome-packaging protein involved in phage assembly. Another phylogenetic tree was constructed using characterized large terminase subunit sequences [38]. As shown in Figure 4B, TerL (ORF14) of the JSSK01 phage clustered within the 3’cos HK97 terminase group. Notably, all members of the “*K1glikevirus*” genus identified in the previous analysis were also found to possess 3’cos HK97 terminase sequences.

### 2.9. JSSK01 Endo-N-Acetylneuraminidase (ENgase; ORF 40) Phylogeny

Phylogenetic analyses based on the whole genome and TerL proteins positioned the K1G group, which included JSSK01, separate from the K1E and K1F groups. To identify the specific K1 capsule type targeted by JSSK01, the domain information of ORF40 (encoding a tail spike protein with an ENgase domain) was examined. The tail spike protein sequence of JSSK01 was compared with those of the K1E, K1F, K1G, and K1H phages. Sequence alignment revealed significant homology with the K1G and K1H phages, with 97.76% and 96.54% identity, respectively. In contrast, the K1F and K1E phages were distantly related, with 47.62% and 45.58% homology of the tail spike proteins, respectively. An additional phylogenetic analysis focusing on ENgase confirmed these findings. The analysis grouped JSSK01 with other K1-dependent phages, similar to K1G and K1H, indicating that the JSSK01 tail spike protein targets the K1 capsule (Figure 4C).

### 2.10. E. coli CPS Screening

To identify K1-harboring *E. coli*, 85 strains were randomly selected and screened via PCR targeting the *kpsMTII* transporter gene, an essential K1 CPS gene. Among the tested strains, 27 yielded a PCR product of the expected size (272 bp), indicating the presence of the *kpsMTII* gene (Table S2 and Figure S1). These strains were further screened to differentiate between R1 and R4 lipopolysaccharide (LPS) types. PCR was performed via primers targeting the *waaL* gene for R1-type LPS and the *waaX* gene for R4-type LPS. All K1-positive strains exhibited amplified products for R1-type LPS with an amplicon size of 551 bp, whereas no specific amplification was observed for R4-type LPS.

### 2.11. JSSK01 Host Sensitivity

A host range test was conducted on all 27 K1-positive bacterial strains to assess the ability of JSSK01 to infect them. The test revealed susceptibility to JSSK01 among 16 strains. Thirteen of these strains were identified as UPEC, and two were classified as MDR. Notably, one MDR strain (18h1k) responded to phage infection but did not form visible plaques on the bacterial lawn (Figure S2 and Table S2). JSSK01 infected 59.3% of all the bacterial strains tested, as characterized by the presence of K1 CPS and R1 LPS. However, some K1 CPS-positive and R1 LPS-positive strains appeared to be resistant to JSSK01.

### 2.12. Therapeutic Potential of JSSK01 in a Zebrafish Model

**Figure 5:**
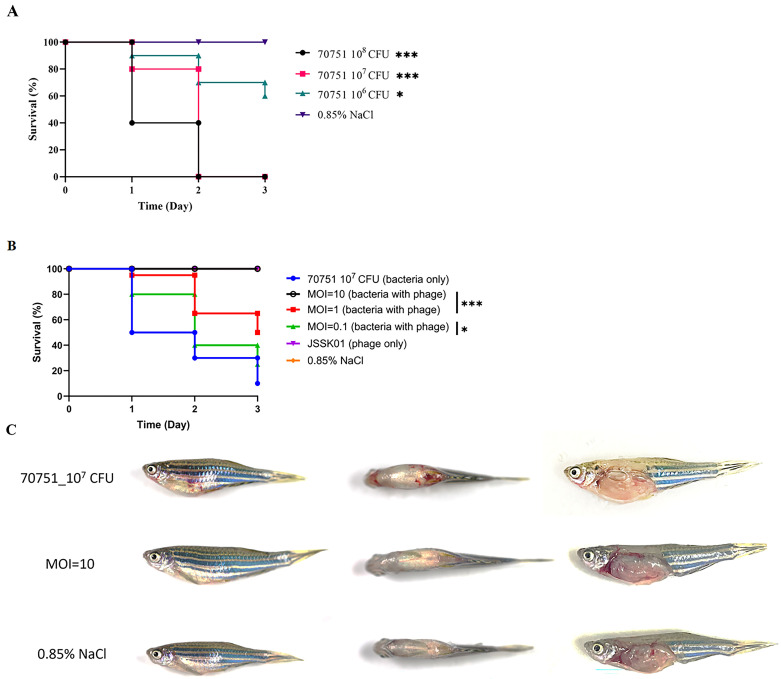
JSSK01 can rescue MDR 70751-infected zebrafish. (**A**) A lethal dose assessment was conducted on three groups of adult zebrafish, each comprising 10 fish. These groups were subjected to intraperitoneal injections of MDR 70751 at various concentrations (10^8^, 10^7^, and 10^6^ CFU in 20 µL of media, as well as 0.85% NaCl as the negative control, represented by the black, red, green, and purple lines, respectively). (**B**) Zebrafish were subjected to JSSK01 treatment (MOI = 10, 1, 0.1) for 30 min after bacterial infection with 10^7^ CFU (black line, red line, green line). Additionally, a group received JSSK01 treatment alone (2.5 × 10^7^ CFU/20 µL, purple line), whereas a negative control group received 0.85% NaCl (orange line). (**C**) The manifestation of disease symptoms in zebrafish infected with *E. coli* 70751 alone was compared with that in the groups rescued by the phage (MOI = 10) and the control group (0.85% NaCl). The views provided are as follows: left, side view; middle, top view; and right, abdominal anatomy. Kaplan–Meier Survival curves were plotted, with the *X*-axis denoting days post-infection and the *Y*-axis indicating the survival percentage. Statistical analysis was conducted via log-rank and generalized Wilcoxon tests in GraphPad Prism 9 software, where *** and * indicate *p* < 0.001 and *p* < 0.05, respectively.

As the lysis test revealed that JSSK01 has significant bactericidal activity, a zebrafish rescue assay was used to test whether JSSK01 could effectively treat K1 *E. coli* infections. First, bacterial counts ranging from 10^6^ to 10^8^ were tested to determine the half-lethal dose, and 10^7^ was found to be the optimal dose (Figure 5A). Zebrafish were subsequently infected with this dose via intraperitoneal injection and treated with JSSK01 at multiplicities of infection (MOI) of 10, 1, and 0.1, respectively, 30 min post-infection. Among the phage-treated groups, the MOI = 10 group exhibited the most effective therapeutic outcome, achieving 100% survival after 3 d. The MOI = 1 group showed 95% survival on the second day and 65% survival by the third day. In the MOI = 0.1 group, no mortality occurred on the first day, with 80% survival observed on the second day, which declined to 40% by the third day (Figure 5B). The results indicated that the phage-treated groups, along with the 0.85% NaCl control group, exhibited no symptoms of disease (Figure 5C). In contrast, the untreated group displayed a 60% survival rate on the first day, with affected fish showing systemic symptoms, such as abdominal swelling and bleeding in the gills, eyes, and other organs. By the third day, the survival rate in the untreated group had declined to 10% (Figure 5B), significantly lower than that in the phage-treated groups. All differences between treated and untreated groups were statistically significant. These findings indicate that JSSK01 has strong therapeutic potential and may serve as an alternative to antibiotics for treating K1 *E. coli* infections.

### 2.13. Functional Assessment of Expressed ENgase (ORF 40)

**Figure 6:**
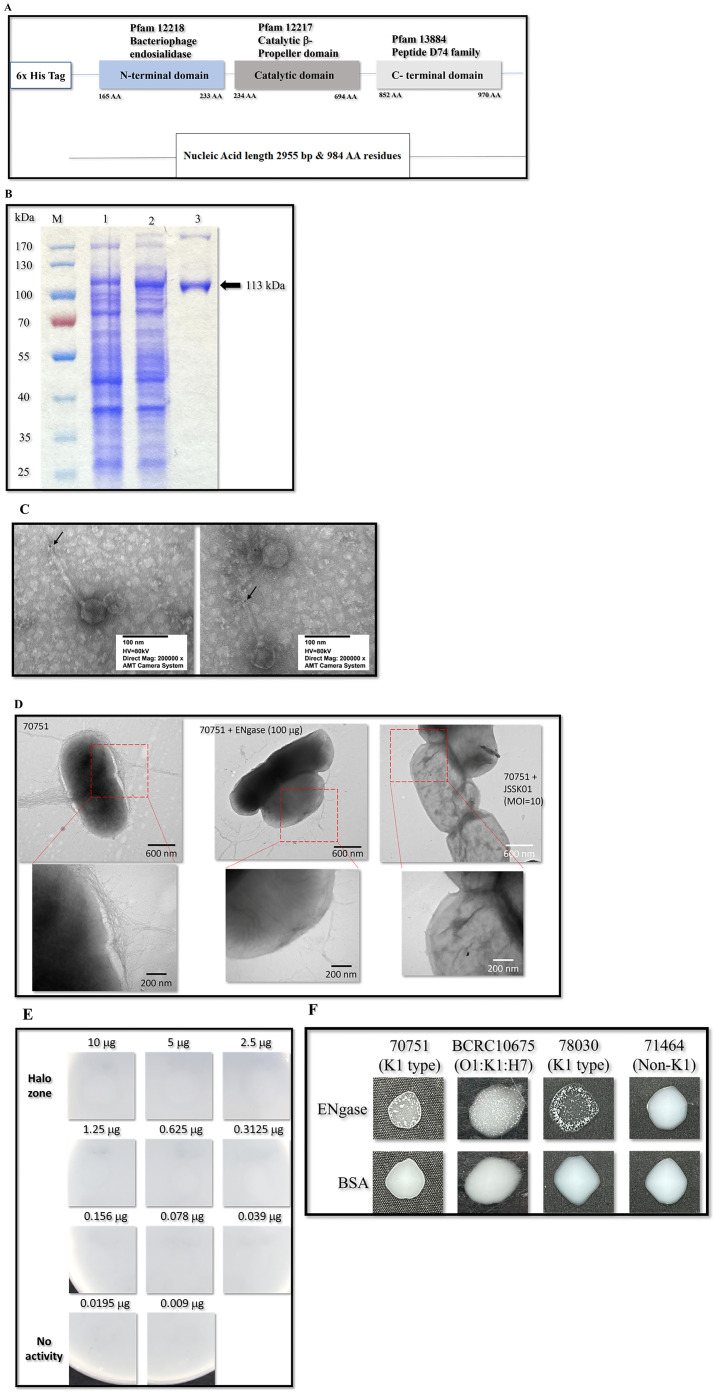
Characterization and functional evaluation of ENgase (ORF40). (**A**) Domain prediction of ENgase via NCBI BLASTp and conserved domain database searches revealed the different functional domains within the protein, indicated by their respective Pfam numbers above. The domains are as follows: the N-terminal extension of bacteriophage endosialidase (Pfam 12218), the catalytic beta propeller domain of bacteriophage endosialidase (Pfam 12217), the unspecified catalytic domain of bacteriophage endosialidase (Pfam 12219), and the peptide S74 family C-terminal domain (Pfam 13884). The tail spike is distinctly marked to denote its unique role. (**B**) Expression of recombinant ENgase in BL21 host cells, where lane M represents the protein marker, lane 1 represents non-induction, lane 2 represents the induction of protein expression, and lane 3 represents the recombinant protein with a predicted size of approximately 113 kDa, as indicated by arrow. (**C**) TEM revealed the localization of ORF40 on the tail of the phage particle, as indicated by arrows. (**D**) Capsule deprivation by ENgase was observed after treatment with recombinant ENgase (middle panels) and phage JSSK01 (right panels) compared with that in untreated cells (left panels). The red boxes have been magnified to observe the effects of the enzyme (bottom panels). (**E**) Recombinant ENgase caused dose-dependent cell lysis, as indicated by a “halo zone” on the host cell lawn. The purified ENgase was serially diluted two-fold to 0.00975 μg. The halo zone could still be observed at 0.039 μg of the enzyme. (**F**) A single colony agglutination test demonstrated the specific recognition of the *E. coli* K1-type capsule by ENgase. 70751 denotes the host strain for JSSK01; BCRC10675 (Bioresource Collection and Research Center, Taiwan) is an O1:K1:H7 for the K1 capsule control; 78030 is a strain containing both K1-type capsules and R1 lipopolysaccharides and is sensitive to JSSK01; and 71464 represents a non-K1 strain used as a negative control. All tests utilized BAS as the agglutination control.

Previous research has shown that the tail and appendage phages determine their host specificity [39,40,41]. JSSK01 exhibits a high degree of specificity toward K1-type *E. coli* strains. ORF40 in the JSSK01 genome is predicted to encode the tail spike protein. Domain analysis via the NCBI Conserved Domain Database revealed four conserved regions within ORF40. These regions included a domain near the N-terminus, annotated as an N-terminal extension of bacteriophage endosialidase (Pfam 12218); a substantial portion of the central region occupied by a highly conserved catalytic β-propeller domain (Pfam 12217) characteristic of endosialidases; a tail spike domain (Pfam 12219); and a C-terminal domain (Pfam 13884) associated with peptidase activity (Figure 6A). Following recombinant expression and purification of the protein encoded by ORF40 (Figure 6B), specific antibodies were generated against this protein. TEM with gold-conjugated secondary antibodies revealed the localization of gold particles predominantly at the distal ends of the phage tails (Figure 6C). To evaluate the enzymatic activity and specificity of the ORF40 protein, *E. coli* 70751 was treated with purified ORF40 for 1 h. TEM analysis revealed that the capsule was removed from the surface of the bacteria (Figure 6D). Zone inhibition and single-colony agglutination tests for K1 *E. coli* were performed using purified ORF40 (Figure 6E,F). The zone of inhibition assay demonstrated a dose-dependent inhibitory effect of ORF40 on bacterial growth. A single-colony agglutination assay confirmed that ORF40 specifically agglutinates K1-type *E. coli.*

## 3. Discussion

*E. coli* is a commensal bacterium that is usually beneficial to its host [1,2]. However, pathogenic *E. coli* variants critically impact host health and are often resistant to conventional antibiotics [2,22]. As a result, bacteriophages are gaining traction as alternative treatments [22]. In this study, a novel bacteriophage, vB_EcoS_JSSK01, which infects two pathogenic *E. coli* variants, MDR *E. coli* and UPEC, was isolated and characterized. Some of these variants possess K1 CPS and can cause infections, such as meningitis [4,5]. The JSSK01 phage was evaluated as a therapeutic agent, and its tail spike protein was used as a diagnostic marker to detect K1 *E. coli*.

The morphological features of the JSSK01 phage are similar to those of siphophages, with an icosahedral head and a long flexible tail with receptors that are radially attached in a club-shaped orientation to its baseplate (Figure 1B) [8,42,43]; these characteristics classify the phage into the genus “*K1glikevirus*” of the subfamily “*Jerseyvirinae*” [44]. Phages classified under the “*Jerseyvirinae*” subfamily are known to infect *Salmonella* spp. and *Escherichia* spp., with the sole exception of *Serratia* phage [45], and they all exhibit a strict lytic cycle [44,45]. The genomic properties of JSSK01 were consistent with those of other *E. coli* phages classified under “*K1glikevirus*” of the subfamily “*Jerseyvirinae*” [44], and this finding was further supported by phylogenetic analysis of JSSK01 ORF14 or the terminase large subunit (TerL) of “*K1glikevirus*”, which have genome packaging systems functioning like 3’cos (HK97) terminases (Figure 4A,B). Although phages classified under the genus “*K1 glikevirus*” are known to infect *E. coli* with K1 CPS, they can be further classified into K1-dependent and K1-independent sub-types based on their tail spikes [46]. K1-independent phages, such as K1ind1, K1ind2, and K1ind3, are known to show endo-*N*-acetylglucosaminidase activity in their tail spikes and belong to the pectatease 3 family (pfam: PF12708). They degrade a specific O-antigen of the *E. coli* serotype [47]. Conversely, K1-dependent phages, such as JSSK01, K1G, and K1H, exhibit ENgase or endosialidase activity in their tail spikes, which helps degrade K1 CPS, a homopolymer linked with α2,8-linked sialic acid residues [46,47]. These tail spikes contain a C-terminal peptidase_S74 domain (PF13884), and the presence of a domain in K1 *Podoviridae* phages is essential for the function of autocleavage, which is crucial for the folding and assembly of mature proteins [48]. Therefore, we meticulously compared the functional protein ORF40 of JSSK01 with the ENgase found in other K1-dependent phages, such as myophage phi92 and podophages K1E and K1F, as well as the siphophages K1G and K1H (Figure 4C). This comparison strongly suggested that JSSK01 is indeed a K1-dependent phage. As a result, JSSK01 was classified into the order Caudovirales, family *Siphoviridae*, subfamily *Jerseyviridae*, genus *K1glikevirus*, and subcategory K1-dependent phage.

When examining the lytic ability of phage JSSK01 across a range of MOIs, it was observed that higher MOIs (100, 10, and 1) rapidly inhibited bacterial growth but eventually showed some indications of phage resistance as the experiment progressed. Conversely, at lower MOIs (0.1 and 0.01), bacterial growth was consistently limited, displaying a linear inhibition with minimal or no signs of resistance (Figure 2C). This sustained inhibition at lower MOIs was attributed to the significant release of phage particles (~123 PFU per infected cell) (Figure 2B), which was balanced with the bacterial population, thus minimizing the development of resistance.

These findings are consistent with previous studies on K1-capsulated *E. coli* phages that highlighted differences in lysis efficiency between K1-dependent (K1-dep) and K1-independent (K1-ind) phages, particularly in the serum. The K1 capsule acts as a protective barrier against phage infection, and successful penetration of the capsule is critical for effective treatment outcomes. Notably, the K1-dep phage showed significantly higher lysis efficiency in serum than the K1-and phage. This enhanced performance can be attributed to the endosialidase activity found in K1-dep phage, an enzyme that degrades the K1 capsule, allowing the phage to gain access to the bacterial surface. Furthermore, supplementation of serum with exogenous endosialidase (endo-N) significantly increased the lysis efficiency of the K1-ind phage, emphasizing the importance of this enzyme in increasing phage effectiveness in serum.

These results highlight the importance of selecting phages based on their ability to balance lytic activity and resistance management, particularly in a therapeutic context. For K1-capsulated bacterial infections, phages with endosialidase activity or adaptability to physiological conditions, such as serum, could enhance therapeutic efficacy and optimize clinical outcomes [46].

The therapeutic potential of JSSK01 was assessed in a zebrafish model infected with MDR70751. Compared with bacteria alone, JSSK01 administration at an MOI of 10 significantly reduced infection risk and resulted in no mortality. No pathological differences were observed compared with those in the NaCl-injected control group (Figure 5B,C). The JSSK01 phage titers remained stable at low temperatures and under alkaline conditions (Figure 2D,E), indicating potential for storage and use in various environments. However, the absence of phage activity at pH 3 suggests that JSSK01 is optimized for environments outside the intestinal tract, where the causative bacteria colonize the host.

Genome comparison of JSSK01 with the closely related siphophages, vB_EcoS_XY1 and K1G, revealed that a wide range of protein-encoding regions was involved in phage recognition by tail spike proteins, such as endo-*N*-acyl-neuraminidase (ORF 40), class I and class II holins (ORFs 78 and 79), terminase for genome packaging (ORF 14), and hallmark genes, such as the NinH-like domain protein (pfam:PF06322) (ORF 11) [44], and a number of hypothetical proteins, remained intact, underscoring their critical importance for phage infectivity and replication in the host (Figure 3A,B, Table S1).

Phages harboring ENgase are known to infect K1 *E. coli* [44]. We observed that JSSK01 infects a broad range of MDR *E. coli* and UPEC strains that carry K1 CPS. Most of these strains also express R1 LPS. In a mutational study aimed at understanding phage–host interactions, mutations were introduced into R1 LPS genes (*waaO* or *waaT*). These mutations affected the ability of a myophage, PNJ1809-36, which also carries endo-*N*-acetylneuraminidase, to form plaques [49]. This led to the proposal that R1 LPS might act as a secondary receptor for complete phage adsorption. Our observations involve phages harboring endo-*N*-acetylneuraminidase; thus, it is plausible that R1 LPS serves as a secondary receptor for these phages as well. However, we could not fully verify this hypothesis. Notably, one *E. coli* strain, 18h1k, which has a K1 CPS but lacks R1 LPS, still exhibited phage sensitivity or depolymerase activity when exposed to a high phage concentration of 10^10^ PFU on the bacterial lawn (Table S2 and Figure S2). This observation supports the hypothesis that R1 LPS acts as a secondary receptor for successful phage infection.

PCR revealed that some *E. coli* strains were positive for K1 CPS but were resistant to JSSK01 infection. This could be explained by the binding of the phage tail spike protein or ENgase to the homopolymer of polysialic acid, which hydrolyzes the α2,8 linkage on K1 CPS and then binds to the secondary receptor or R1 LPS for full adsorption [49]. Interference at first binding may not have occurred between *E. coli* strains subjected to JSSK01 infection. We suspect that these *E. coli* strains had previously undergone similar natural phage stress before being isolated from the source. As a result, the bacterial CPS might have undergone phase variation, resulting in phage interactions [50].

Additionally, there might be misidentification in screening *E. coli* K1 CPS from similar strains, such as K92 and K24. In this study, the identification of K1 *E. coli* was based on the presence of a conserved transporter gene, *kpsMTII*, involved in K-antigen translocation to the bacterial cell surface. Strains, such as *E. coli* K92, have a polysialic acid arrangement with either an α2,9 linkage or alternating α2,8 and α2,9 linkage [51], similar to K1 CPS, indicating the phenotypic relatedness of CPS and suggesting genetic relatedness with the genes involved in capsular synthesis. Therefore, we assume that PCR screening to detect K1 CPS might not be entirely successful and that misidentification could occur for similar *E. coli* strains due to genetic relatedness at the *kps* cluster. Furthermore, in silico PCR was performed using the *kpsMTII* primer set on the collected *kps* cluster sequences of *E. coli* K1, K92, and K24, which showed possible amplification (Figure S3). Thus, bacterial strains defined as K1-related but not susceptible to JSSK01 infection and not agglutinated by ENgase might not be true K1 *E. coli* strains.

Although molecular screening of K1 *E. coli* was not entirely successful, evaluation with recombinantly expressed JSSK01 ENgase successfully differentiated K1 *E. coli* from K1-related strains. This was confirmed by a conjugation assay, where Ni-NTA beads coated with purified ENgase agglutinated when introduced into K1 *E. coli*, indicating cross-reactivity between K1 CPS and ENgase. Testing under similar conditions on the *E. coli* strain, BCRC10675, with a phenotype of O1:K1:H7 was positive for agglutination (Figure 6D), confirming that the JSSK01 phage and its tail spike protein (ENgase) interact with K1 CPS for successful phage infection.

This study suggests that JSSK01 is a potential alternative for treating MDR extraintestinal *E. coli* infections, particularly those caused by K1 capsular *E. coli* strains. Additionally, the ORF40 tail protein can be recombinantly expressed for specific K1 CPS recognition, serving as a diagnostic tool for identifying K1 capsular *E. coli.*

## 4. Materials and Methods

### 4.1. Bacterial Growth Conditions

Bacterial strains, including the indicator host strain MDR 70751, were clinical isolates obtained from Buddhist Tzu Chi Hospital, Hualien, Taiwan, and cultured in LB broth and LB agar plates (Bio Basic Inc., Armonk, NY, USA) and incubated at 37 °C for 16 to 18 h.

### 4.2. Antibiotic Susceptibility Test for MDR E. coli and UPEC Strains

Antibiotic susceptibility tests were conducted via the agar disc diffusion method on four phage-sensitive bacterial strains: 70751, 78030, G0032, and G0218 (Table 1) [52]. The strains were cultured overnight and adjusted to an OD_600_ of 0.1 in 1 mL of 0.85% NaCl buffer. The bacterial cultures were streaked onto Mueller–Hinton agar plates. Antimicrobial susceptibility test discs containing ciprofloxacin [5 μg], kanamycin [30 μg], erythromycin [15 μg], tetracycline [30 μg], colistin [30 μg], chloramphenicol [30 μg], imipenem [10 μg], meropenem [10 μg], ertapenem [10 μg], and gentamycin [10 μg] (BD BBL™ Sensi-Disc™, Singapore) were placed on the plates and incubated at 37 °C for 16–18 h. The diameter of the inhibition zone was compared with reference values to determine the antibiotic sensitivities. *E. coli* BCRC11633 (ATCC 8739) (Bioresource Collection and Research Center, Taiwan) was used as a quality control strain. The diameters were classified on the basis of the Clinical and Laboratory Standard Institute guidelines [52].

### 4.3. Phage Isolation and Purification

Wastewater samples collected from Tzu Chi Hospital were prefiltered through a 0.45-µm membrane to remove debris. To isolate *E. coli* phages, a bacterial cocktail was prepared by mixing 200 µL of five different *E. coli* clinical isolated strains, resulting in a total volume of 1.0 mL. This bacterial mixture was mixed with the filtered wastewater sample and incubated at 37 °C with shaking for 72 h. Samples were collected every 24 h and centrifuged, and overlay agar assays were performed to identify phage-sensitive bacterial strains, as previously described [53]. The enrichment of phages involved three rounds of infection with the host strain MDR 70751, followed by amplification in 100 mL bacterial cultures. The phages were purified by sorting the cell debris via membrane filtration and centrifugation. The pelleted phage particles were suspended in 1 mL of SM buffer (0.05 M Tris–HCl (pH 7.5), containing 0.1 M NaCl, 0.008 M MgSO4•7H_2_O, and 0.01% gelatin), further purified via the CsCl density gradient method, and desalinated via dialysis [54,55].

### 4.4. TEM

For morphological characterization, 10^9^ PFU/mL purified phage particles were adsorbed onto a formvar-coated copper grid (300 mesh; Electron Microscopy Sciences, Hatfield, PA, USA) [56]. The particles were then negatively stained with 2% uranyl acetate and observed via a Hitachi H-7500 transmission electron microscope (Hitachi Co., Tokyo, Japan) operated at 80 kV and located at the TEM facility of Tzu Chi University.

### 4.5. Adsorption, One-Step Growth Curve, and Lysis Curve

The host strain was grown to the mid-log phase and infected with JSSK01 phage lysate at an MOI of 0.0005 [57]. The mixture was incubated on ice for 20 min. Samples were collected every 2 min and centrifuged, and the supernatant was serially diluted to quantify unabsorbed phages via a plaque assay. The adsorption efficacy was calculated on the basis of the initial and unabsorbed phage titers. Similarly, a one-step growth experiment involved culturing the host strain to mid-log phase in LB media, infecting it with phage lysate at an MOI of 0.01, and incubating it on ice for 20 min. After centrifugation, the pellet was resuspended in fresh LB medium and transferred to a large volume (50 mL) of medium for incubation. Samples were collected every 5 min, serially diluted, and subjected to plaque assays to determine the number of phage particles released per infected cell (burst size). Phage lytic activity was assessed by infecting the host strain with different phage concentrations (MOIs of 100, 10, 1, 0.1, and 0.01) in a microtiter plate for 12 h at 37 °C, and OD_600_ measurements were taken every 30 min to monitor bacterial growth and lysis. All experiments were performed in triplicate.

### 4.6. Thermal and pH Stability Assays

The phage lysate was adjusted to a titer of 10^8^ PFU/100 µL and mixed with 900 µL of LB broth at neutral pH or adjusted to various pH values. To assess thermal stability, the mixture was exposed to different temperatures (25, 37, 50, and 65 °C) for 1 h at a neutral pH [58]. To assess pH stability, the mixtures were exposed to different pH values (3, 5, 7, 9, and 11) for 1 h at 37 °C. The phage titers were determined via a plaque assay. All experiments were performed in triplicate.

### 4.7. Structural Protein Analysis

Purified phage lysate (1.17 × 10^11^ PFU/20 µL) was mixed with 5 µL protein sample buffer (100 mM Tris-HCl at pH 6.8, 4% sodium dodecyl sulfate (SDS), 0.2% bromophenol blue, and 20% glycerol), boiled for 15 min, and then separated via SDS–polyacrylamide gel electrophoresis (SDS–PAGE) via a 12% polyacrylamide gel [59]. The gels were stained with Coomassie Brilliant Blue R250 solution (0.1% Coomassie R250, 10% acetic acid, and 40% methanol) for 3 h and then destained with 20% methanol and 10% acetic acid for 1 h. Band images were captured using a Thermo iBright FL1500 Imaging System (Thermo Fisher Scientific Inc., Waltham, MA, USA). Major coat protein bands were excised from the gel and subjected to MS/MS analysis for protein identification. The resulting protein sequences were identified by comparison with an in-house database maintained by the Advanced Instrumentation Center of the Department of Medical Research, Hualien Tzu Chi Hospital.

### 4.8. Genome Sequencing and Analysis

Genomic DNA was extracted from CsCl-purified phage particles (5 × 10^10^ PFU/500 µL) using a commercially available kit (Norgen BioTek Corp., Thorold, ON, Canada). Phage genome sequencing was performed by BIO TOOLS Co., Ltd. (Taipei, Taiwan). Briefly, library construction was carried out via a NEBNext^®^ DNA Library Prep Kit (New England Biolabs, Ipswich, MA, USA). Following quality control of the constructed library, paired-end sequencing was performed on an Illumina high-throughput sequencing platform (Illumina, San Diego, CA, USA). The assembled genome sequences were annotated via the RAST pipeline within RASTtk [60,61,62]. Putative homologs of the phage genomes were identified via the National Center for Biotechnology Information (NCBI) basic local alignment search tool for nucleotides (BLASTn), and protein functions were predicted via protein BLAST (BLASTp) and position-specific iterated BLAST (PSI-BLAST) (https://blast.ncbi.nlm.nih.gov/Blast.cgi (accessed on 23 November 2021)). Conserved protein domains within annotated ORFs were identified via the NCBI Conserved Domain Database [63,64,65]. The phage genome was analyzed and organized via SnapGene Viewer (https://www.snapgene.com/snapgene-viewer, accessed on 23 November 2021) and compared with other similar phage genomes via Easyfig [66]. The phylogenetic relationships between the JSSK01 terminase large subunit (TerL) and ENgase protein sequences were inferred via multiple sequence alignment via ClustalW followed by neighbor-joining tree construction with 1000 bootstrap replications via MEGA 11 [67]. A broader phylogenetic analysis of the phage genome was performed via the VICTOR platform (https://ggdc.dsmz.de/victor.php, accessed on 23 November 2021) [68]. The complete genome sequence of JSSK01 has been deposited into the NCBI GenBank database under accession number OQ442786.

### 4.9. K1 CPS Screening

PCR was performed to screen *E. coli* strains for the presence of the K1 CPS transporter gene (*kpsMTII*). The specific primers and PCR conditions used have been previously described [10]. The PCRs involved initial denaturation at 95 °C for 12 min, followed by 25 cycles of denaturation at 94 °C for 30 s, annealing at 58 °C for 30 s, extension at 72 °C for 3 min, and a final extension step at 72 °C for 10 min. The amplified DNA products, with an expected size of 272 base pairs (bp), were visualized on a 2% agarose gel stained with ethidium bromide. The PCR products were sequenced and compared with reference sequences via BLASTn.

### 4.10. R1 and R4 LPS Screening

R1 and R4 core LPS were screened via two divergent primer sets, R1 (*waaL*) and R4 (*waaX*), obtained from a previous study [69], with PCR conditions including a change in the annealing temperature from 50 °C to 52 °C, initial denaturation at 94 °C for 1 min, followed by 35 amplification cycles of denaturation at 94 °C for 20 s, annealing at 52 °C for 30 s, extension at 72 °C for 2 min 15 s, and a final extension at 72 °C for 2 min, with the amplified PCR product visualized via 1% agarose gel electrophoresis with ethidium bromide staining.

### 4.11. Zebrafish Infection and Phage Rescue

Owing to its genetic, anatomical, and physiological similarities to mammals, the zebrafish (*Danio rerio*) has become a popular model organism for behavioral, genetic, and infectious disease research [70]. Consequently, this study utilized a zebrafish model infected with MDR 70751 to evaluate the efficacy of phage JSSK01. The fish were maintained at the Tzu Chi University Fish Core Facility following established protocols [30]. Briefly, a mixed population of adult male and female zebrafish were housed together in 9-L tanks at 28 °C under a 14-h light/10-h dark cycle. The infection experiments were adapted from previously described methods [58], with some modifications.

Adult zebrafish were divided into groups of 10 fish each. One group was anesthetized with 0.2% tricaine and received an intraperitoneal injection of 20 μL (2.5–4 × 10^7^ CFU) of the *E. coli* strain MDR 70751 suspended in 0.85% NaCl via a 32 G needle. Another group received the same dose of *E. coli*, followed by an intraperitoneal injection of 2.5 × 10^8^ PFU JSSK01 (MOI = 10) 30 min later. Additionally, to assess the potential acute toxicity of JSSK01, a separate group of zebrafish was injected with 2.5 × 10^8^ PFU JSSK01 or 0.85% NaCl alone without bacterial infection. Following treatment, the zebrafish were transferred to separate tanks and monitored for survival at least five times daily for 3 d. The number of surviving fish in each group on day three was recorded, and statistical analysis (Fisher’s exact test) was performed to evaluate the therapeutic efficacy of JSSK01. Kaplan–Meier survival curves were generated via GraphPad Prism 9 software (GraphPad, La Jolla, CA, USA) to determine survival rates over 3 d. Statistical comparisons of survival curves were performed via the log-rank test or the generalized Wilcoxon test. The animals were maintained and handled in accordance with the recommendations of the Guide for the Care and Use of Laboratory Animals (2021). All animal experiments were conducted by trained personnel and approved by the Institutional Animal Care and Use Committee (IACUC) of Tzu Chi University, Taiwan (IACUC Approval No. 111091). Two independent experiments were performed.

### 4.12. Expression and Purification of ENgase

The ORF40 gene, which encodes ENgase, was cloned and inserted into the pGEM-T Easy vector (Promega Corporation, Madison, WI, USA) and subsequently cleaved at the *Bam*HI and *Xho*I sites before being ligated into the pET30a expression vector (Novagen, WI, USA). The detailed procedures and primer sequences are shown in Table S3. The resulting recombinant plasmids containing ORF40 within pET30a were plated on LB plates containing kanamycin. The resulting colonies were verified via PCR and sequencing. BL21 (DE3) cells containing the pET30a_*orf40* plasmid were cultured, induced for protein expression, and purified via a nickel–nitrilotroacetic acid (Ni–NTA) column (Bio-Rad Laboratories, Inc., Hercules, CA, USA) according to the manufacturer’s instructions. The purified protein was quantified via a BCA kit (Thermo Fisher Scientific) according to the manufacturer’s instructions.

### 4.13. Determination of the Anti-Capsule Activity of ENgase

The ability of recombinant ENgase to degrade K1 CPS was assessed via a previously described method for depolymerase activity [71]. Briefly, various concentrations of purified ENgase were spotted on a lawn of the host strain, *E. coli* MDR 70751. Following incubation with 100 ng/μL depolymerase solution at 37 °C for 6 h, morphological alterations were observed via TEM.

### 4.14. Direct Colony Latex Agglutination Assay

The ability of ENgase to bind to *E. coli* was assessed via a latex agglutination assay performed as previously described [71]. Briefly, 10 µL of a suspension containing 0.6% polystyrene sulfate latex beads (0.8 µm; Thermo Fisher Scientific) coated with either purified ENgase or bovine serum albumin was spotted onto a clean glass slide. A single, isolated bacterial colony from a freshly grown culture on an LB agar plate was then mixed with the spotted bead suspension. The slide was gently swirled to disperse any agglutinated clumps within the spotted radius. The results were observed within 1 to 2 min of incubation at room temperature. The aggregation of bacteria around the spotted beads indicated a positive interaction between ENgase and the *E. coli* cells, whereas the lack of agglutination indicated a negative interaction.

### 4.15. Statistical Analysis

The data were analyzed via appropriate statistical methods, depending on the experimental design. For count data, a log transformation with a constant value of 1 added was performed to achieve approximate normality and avoid taking logarithms of zero. Significance was set at *p* < 0.05, and pairwise comparisons between groups were performed using t-tests. For zebrafish survival data, Kaplan–Meier curves were generated using GraphPad Prism 9 to depict the cumulative probability of survival over 3 d. Statistical comparisons of survival curves were performed using the log-rank test or generalized Wilcoxon test, where ***, **, and * indicate *p* < 0.001, *p* < 0.01, and *p* < 0.05, respectively.

## 5. Conclusions

In this study, we isolated a novel K1-dependent bacteriophage, JSSK01, which could infect MDR *E. coli*. Its host range was specific to K1 *E. coli,* and it could be used for therapy as a phage cocktail. The tail spike protein could be used for specific detection of K1 *E. coli* strains, potentially reducing the diagnosis time under optimized conditions. Our future studies will characterize ORF40 further to refine its ability to detect K1 capsules and explore its potential therapeutic applications in bacterial infections. Additionally, we plan to investigate its compatibility with other treatments to increase its effectiveness in combating infections.

## Figures and Tables

**Table 1:** Antibiotic susceptibility of phage-sensitive bacterial strains.

Antibiotics	Bacterial Strains							
	G0032		G0218		78030		70751	
Ciprofloxacin	^a^ 36	^b^ S	39	S	6.5	R	10	R
Kanamycin	23	S	24	R	18.5	S	23	S
Erythromycin	12	S	13	S	6.5	R	0	R
Tetracycline	7.5	R	25	S	11	R	11	R
Colistin	22	S	31	S	32	S	14	S
Chloramphenicol	14	I	13	I	14	I	30	S
Imipenem	29	S	28.5	S	23	S	29	S
Meropenem	28.5	S	30	S	28	S	30	S
Ertapenem	31	S	32	S	29.5	S	32	S
Gentamycin	22	S	20	S	6.5	R	0	R

^a^: Diameter of the inhibition zone in mm; ^b^: susceptibility to antibiotics; S: sensitive; R: resistant; I: intermediate (mildly insensitive).

## Data Availability

All the data generated or analyzed during this study are included in this published article and its Supplementary Information Files. The complete genome sequence of JSSK01 has been deposited into the NCBI GenBank database under accession number OQ442786.

## Supplementary Materials

The following supporting information can be downloaded at: https://www.mdpi.com/article/10.3390/ijms252312497/s1.
