# Supplementary material for: Therapeutic and Diagnostic Potential of a Novel K1 Capsule Dependent Phage, JSSK01, and Its Depolymerase in Multidrug-Resistant Escherichia coli Infections
Source: Int J Mol Sci. 2024 Nov 21;25(23):12497. doi: 10.3390/ijms252312497 (PMC11641727; doi:10.3390/ijms252312497)
Supplement: Supplementary file 1 [file ijms-25-12497-s001.zip › figure caption.pdf]

Figure S1: Bacterial polysaccharide screening. Both MDR and UPEC strains are screened for the *kpsMTII* gene for K1 CPS identification.

Figure S2: Spot test to identify bacterial sensitivity to JSSK01 phage infection. a) Clear zones were detected upon spotting 10^8 and 10^9 PFU phage titers on MDR 78030 bacterial lawn. b) 18h1k strain displayed weaker lysis due to lack of R1 LPS.

Figure S3: In *silico* PCR amplification of *kpsM* gene. K1(X53819), K92 (MG739441), and K24 (MG739439) *E. coli kps* gene clusters were retrieved from the NCBI database. In *silco* PCR amplification was performed by SnapGene using *kpsM* primers sets that are used to detect K1 CPS detection. All three *E. coli* strains were positive for *kpsM* gene with a 271 bp PCR product.
