# Supplementary material for: Therapeutic and Diagnostic Potential of a Novel K1 Capsule Dependent Phage, JSSK01, and Its Depolymerase in Multidrug-Resistant Escherichia coli Infections
Source: Int J Mol Sci. 2024 Nov 21;25(23):12497. doi: 10.3390/ijms252312497 (PMC11641727; doi:10.3390/ijms252312497)
Supplement: Supplementary file 1 [file ijms-25-12497-s001.zip › Table S1.pdf]

Table S1. JSSK01 phage annotation with predicted protein function

ORF	Start	End	Strand	Amino acid length	Annotated function	Conserve Domain and PSI-BLAST	Query Cover	Identity	Accession Number
ORF-01	350	547	+	65	Hypothetical protein	head protein [Escherichia phage NTEC3]	100%	100%	QIG59231.1
ORF-02	547	696	+	65	hypothetical protein [Siphoviridae sp.]	N/D	100%	100%	DAK06957.1
ORF-03	693	902	+	69	Hypothetical protein	N/D	100%	100%	QIG59229.1

ORF-04	899	1096	+	65	Hypothetical protein	N/D	100%	100%	QIG59228.1
ORF-05	1093	1251	+	52	DUF2737 family protein [Escherichia coli]	DUF2737 superfamily [Non-specific hit] pfam10930	100%	96.15%	WP_193017843.1
ORF-06	*1248*	*1421*	+	*57*	Hypothetical protein	Nin protein [Escherichia phage vB_EcoS_XY2]	100%	59.65%	YP_009821897.1
ORF-07	1414	1704	+	96	Hypothetical protein [Siphoviridae sp.]	DUF551 domain-containing protein [Salmonella bongori]	100%	96.88%	DAO31735.1
ORF-08	1691	1873	+	60	Hypothetical protein TE1_068 [Escherichia phage	N/D	100%	98.33%	QNO11932.1

					vB_EcoS_fTaEco 01]				
ORF-09	1960	2130	+	56	TPA: hypothetical protein [Escherichia coli]	N/D	100%	100%	HAM5207844. 1
ORF-10	2127	2324	+	65	TPA: MAG TPA: protein of unknown function DUF4969 [Siphoviridae sp.]	TPA: MAG TPA: FtsZ family, C-terminal domain [Bacteriophage sp.]	100%	46.15%	DAG81214.1
ORF-11	2321	2557	+	78	transcriptional regulator [Escherichia phage vB_EcoS_XY1]	Phage_NinH superfamily [Non-specific hit] pfam06322	100%	98.72%	QIG59224.1

ORF-12	2554	2655	+	33	hypothetical protein GT371_00032 [Escherichia phage vB_EcoS_XY1]	N/D	100%	100%	QIG59223.1
ORF-13	2777	3289	+	170	putative terminase small subunit [Escherichia phage vB_EcoS_XY1]	N/D	100%	100%	QIG59222.1
ORF-14	3279	4550	+	423	terminase large subunit	Terminase_6 superfamily (pfam03237)	100%	100%	YP_009821838.1

					[Raoultella phage RP180]				
ORF-15	4563	6044	+	493	TPA: MAG TPA_asm: portal [Siphoviridae sp.]	DUF4055 superfamily [Non-specific hit] pfam13264	100%	97.57%	DAL40193.1
ORF-16	6114	7157	+	347	TPA: MAG TPA: minor capsid protein [Siphoviridae sp.]	Phage_Mu_F superfamily [Non-specific hit] (PSSMID 213641)	100%	95.68%	DAG81152.1
ORF-17	7157	7618	+	153	tail protein [Escherichia phage vB_EcoS_XY1]	N/D	100%	98.69%	QIG59218.1

ORF-18	7961	8347	+	128	putative spanin [Escherichia phage K1H]	N/D	100%	99.22%	YP_009168838.1
ORF-19	8539	9240	+	233	hypothetical protein [Escherichia coli]	scaffold protein [Escherichia phage vB_EcoS_XY1]	100%	100%	WP_196235762.1
ORF-20	9243	10292	+	349	putative major capsid protein [Escherichia phage vB_EcoS_fFiEco03]	N/D	100%	95.99%	QNO11646.1

ORF-21	10354	10695	+	113	decoration protein [Escherichia phage vB_EcoS_XY1]	N/D	100%	96.46%	QIG59213.1
ORF-22	10732	10911	+	59	head-tail joining protein [Escherichia phage Shashou]	N/D	100%	94.92%	QEA09411.1
ORF-23	10915	11427	+	170	TPA: MAG TPA: putative head-tail connector protein [Bacteriophage sp.]	N/D	100%	96.47%	DAJ10475.1

ORF-24	11430	12044	+	204	putative neck protein [Escherichia phage 26]	N/D	100%	94.12%	UAW06942.1
ORF-25	12044	12403	+	119	head-to-tail connector complex protein [Escherichia phage vB_EcoS_fTaEco03]	N/D	100%	99.16%	QNO11950.1
ORF-26	12400	12795	+	131	TPA: MAG TPA: tail component [Siphoviridae sp.]	N/D	100%	97.71%	DAG23371.1

ORF-27	12795	13208	+	137	TPA: MAG TPA: tail completion protein [Siphoviridae sp.]	DUF4128 superfamily [Non-specific hit] pfam13554	100%	97.08%	DAG24577.1
ORF-28	13211	14377	+	338	tail tube protein [Escherichia phage vB_EcoS_XY1]	Phage_tube_2 [Specific hit] pfam18906	100%	97.16%	QIG59206.1
ORF-29	15077	14406	-	223	TPA: hypothetical protein [Salmonella enterica]	Phage_pRha [Specific hit] pfam09669	100%	97.31%	HAF3913915.1

ORF-30	15427	15197	-	76	hypothetical protein HSE2_gp047 [Escherichia phage vB_EcoS_HSE2]	phosphodiesterase [Escherichia phage Shashou]	100%	98.68%	AUE23516.1
ORF-31	16626	15424	-	400	calcineurin-like phosphoesterase [Escherichia phage vB_EcoS-phiEc4]	N/D	100%	97.25%	QYU43870.1
ORF-32	16911	16786	-	41	TPA: MAG TPA: hypothetical protein [Siphoviridae sp.]	superinfection immunity protein [Escherichia phage Schulenburg]	100%	97.56%	DAE48289.1

ORF-33	16952	17371	+	139	hypothetical protein [Escherichia coli]	phage tail assembly chaperone [Escherichia coli]	100%	99.28%	WP_137500301.1
ORF-34	17470	17736	+	88	putative tail assembly chaperone [Escherichia phage vB_EcoS_fTaEco01]	N/D	100%	100%	QNO11886.1
ORF-35	17729	20002	+	757	TPA: MAG TPA: tail length tape measure protein [Siphoviridae sp.]	PTZ00121 superfamily	100%	98.68%	DAE48230.1

						PTZ00121 (PSSMID 173412) MAEBL; Provisional			
ORF-36	20002	20505	+	167	hypothetical protein KE1_025 [Escherichia phage vB_EcoS_fKuEco01]	putative gene transfer agent protein [Escherichia phage ULINTec2]	99%	97.59%	QNO11740.1
ORF-37	20502	21017	+	171	DUF1833 family protein [Escherichia coli]	DUF1833 superfamily [Non-specific hit] pfam08875	100%	99.42%	WP_137500303.1
ORF-38	21014	21379	+	121	hypothetical protein	peptidoglycan endopeptidase	100%	100%	YP_009168809.1

					[Escherichia phage K1G]	[Escherichia phage phiWAO78-1]			
ORF-39	21442	23925	+	827	TPA: MAG TPA: tail protein [Siphoviridae sp.]	COG4733 superfamily<br>COG4733 (PSSMID 227076) Phage-related protein, tail component	100%	98.31%	DAK06934.1
ORF-40	23938	26892	+	984	endo-N-acetylneuraminidase [Phage #D]	End_beta_propel<br>[Specific hit] pfam12217	100%	97.87%	BAA32990.1
ORF-41	27058	26921	-	45	hypothetical protein [Escherichia coli]	exonuclease subunit [Escherichia phage vB_EcoS_XY1]	100%	100%	WP_196235743.1

ORF-42	27570	27055	-	171	DNA primase [Escherichia phage vB_EcoS_XY1]	TOPRIM_primases [Specific hit] cd01029	100%	98.25%	QIG59265.1
ORF-43	28991	27567	-	474	helicase [Escherichia phage G AB-2017]	HepA [Specific hit] COG0553 (PSSMID 223627)	100%	98.52%	AQN31823.1
ORF-44	29667	28984	-	227	DNA cytosine methyltransferase [Salmonella phage S55]	N/D	100%	98.24%	QMS41866.1
ORF-45	29855	29664	-	63	MULTISPECIES: hypothetical	N/D	100%	100%	WP_076915465.1

					protein [Enterobacteriaceae]				
ORF-46	30173	29886	-	95	VRR-NUC domain protein [Escherichia phage vB_EcoS_HSE2]	VRR-NUC-like [Specific hit] cd22365	100%	97.85%	AUE23475.1
ORF-47	30258	30160	-	32	TPA: MAG TPA: 3-hydroxyanthranilate 3,4-dioxygenase [Siphoviridae sp.]	N/D	100%	96.88%	DAN86871.1

ORF-48	30386	30255	-	43	TPA: MAG TPA: hypothetical protein [Siphoviridae sp.]	TPA: MAG TPA: Rab5-interacting protein (Rab5ip) [Siphoviridae sp.]	100%	95.35%	DAE48299.1
ORF-49	32547	30376	-	723	TPA: DNA polymerase [Escherichia coli]	DNA_pol_A superfamily [Non-specific hit] cd08642	100%	98.89%	HAM5207808.1
ORF-50	33233	32607	-	208	hypothetical protein FE2_044 [Escherichia phage vB_EcoS_fFiEco02]	TPA: MAG TPA: DNA helix destabilizing protein [Siphoviridae sp.]	100%	100%	QNO11605.1

ORF-51	34562	33321	-	413	putative nuclease [Escherichia phage vB_EcoS_fPoEco 01]	Cas4_I-A_I-B_I-C_I-D_II-B superfamily [Non-specific hit] pfam10926	100%	98.55%	QNO11829.1
ORF-52	34717	34559	-	52	hypothetical protein GT371_00064 [Escherichia phage vB_EcoS_XY1]	N/D	100%	100%	QIG59255.1
ORF-53	34863	34714	-	49	hypothetical protein GT371_00063 [Escherichia	N/D	100%	100%	QIG59254.1

					phage vB_EcoS_XY1]				
ORF-54	35132	34860	-	90	hypothetical protein GT371_00062 [Escherichia phage vB_EcoS_XY1]	gp45 [Sodalis phage SO1]	100%	100%	QIG59253.1
ORF-55	35332	35129	-	67	hypothetical protein GT371_00061 [Escherichia phage vB_EcoS_XY1]	N/D	100%	100%	QIG59252.1

ORF-56	35559	35344	-	71	hypothetical protein GT371_00060 [Escherichia phage vB_EcoS_XY1]	N/D	100%	100%	QIG59251.1
ORF-57	36127	35603	-	174	13.88 kDa late protein [Escherichia phage vB_EcoS_XY2]	N/D	100%	79.31%	QIG59283.1
ORF-58	36379	36152	-	75	hypothetical protein GT371_00058 [Escherichia	N/D	100%	100%	QIG59249.1

					phage vB_EcoS_XY1]				
ORF-59	36501	36716	+	71	transcriptional repressor [Escherichia phage vB_EcoS_XY1]	HTH_XRE [Specific hit] cd00093	100%	100%	QIG59248.1
ORF-60	38956	36734	-	740	replicative helicase/primase [Escherichia phage vB_EcoS_XY2]	P-loop_NTPase superfamily [Non-specific hit] cd01125	100%	99.32%	QIG59281.1
ORF-61	39081	38953	-	42	hypothetical protein G_51	NA	100%	100%	AQN31808.1

					[Escherichia phage G AB-2017]				
ORF-62	39441	39151	-	96	hypothetical protein GT372_00012 [Escherichia phage vB_EcoS_XY2]	N/D	100%	100%	QIG59279.1
ORF-63	39620	39438	-	60	helix-turn-helix domain protein [Escherichia phage vB_EcoS_XY2]	HTH_17 [Specific hit] pfam12728	100%	98.33%	QIG59278.1

ORF-64	40102	40320	+	72	hypothetical protein [Escherichia coli]	N/D	100%	100%	WP_196235769.1
ORF-65	40339	40524	+	61	hypothetical protein MONNJMKB_00053 [Escherichia phage vB_EcoS-phiEc3]	N/D	100%	98.36%	QZI78320.1
ORF-66	40524	40709	+	61	TPA: MAG TPA: hypothetical protein [Bacteriophage sp.]	N/D	100%	93.44%	DAH69334.1

ORF-67	40732	40926	+	64	hypothetical protein GT371_00051 [Escherichia phage vB_EcoS_XY1]	N/D	100%	100%	QIG59242.1
ORF-68	40923	41078	+	51	glycosyl hydrolase [Escherichia phage L AB-2017]	N/D	100%	100%	AQN31870.1
ORF-69	41075	41218	+	47	hypothetical protein GT371_00050 [Escherichia	N/D	100%	100%	N/D

					phage vB_EcoS_XY1]				
ORF-70	41222	41422	+	66	valyl-tRNA synthetase [Escherichia phage 590B]	DUF551 superfamily [Non-specific hit] pfam04448	100%	98.48%	QVR48611.1
ORF-71	41557	41727	+	56	TPA: MAG TPA: dextransucrase [Bacteriophage sp.]	N/D	100%	92.86%	DAJ10508.1
ORF-72	41796	41981	+	61	hypothetical protein GT371_00047 [Escherichia	gp82 [Escherichia phage Tls]	100%	98.36%	QIG59238.1

					phage vB_EcoS_XY1]				
ORF-73	42086	42412	+	108	TPA: MAG TPA: langerin binding protein [Siphoviridae sp.]	N/D	100%	77.01%	DAG75867.1
ORF-74	42409	42612	+	67	hypothetical protein [Salmonella phage vB_SpuS_Sp4]	Bacteriophage T7, Gp1.7 [Corchorus olitorius]	100%	98.51%	AWY03011.1
ORF-75	42615	42878	+	87	hypothetical protein GT371_00046	TPA: MAG TPA: Pre-mRNA-splicing factor 8, Pre-mRNA-splicing	100%	97.70%	QIG59237.1

					[Escherichia phage vB_EcoS_XY1]	factor-mRNA splicing, spliceosome, post-catalytic, P [Siphoviridae sp.]			
ORF-76	42881	43111	+	76	hypothetical protein GT371_00045 [Escherichia phage vB_EcoS_XY1]	hNH endonuclease [Escherichia coli]	77%	89.83%	QIG59236.1
ORF-77	43108	43401	+	97	TPA: hypothetical protein [Salmonella enterica subsp.	HNH endonuclease [Klebsiella pneumoniae]	100%	95.88%	HAE0051440.1

					enterica serovar Enteritidis]				
ORF-78	43477	43785	+	102	putative holin-like class II [Escherichia phage vB_EcoS_XY1]	N/D	100%	97.06%	QIG59234.1
ORF-79	43778	44050	+	90	putative holin-like class I [Escherichia phage ST20]	N/D	100%	100%	ASH99343.1
ORF-80	44028	44507	+	160	endolysin [Escherichia	lyz_endolysin_autolysin [Specific hit] cd00737]	100%	96.88%	QYU43915.1

		phage vB_EcoS-phiEc4]	

Note:

N/D **:** Not determined
