# Supplementary material for: Therapeutic and Diagnostic Potential of a Novel K1 Capsule Dependent Phage, JSSK01, and Its Depolymerase in Multidrug-Resistant Escherichia coli Infections
Source: Int J Mol Sci. 2024 Nov 21;25(23):12497. doi: 10.3390/ijms252312497 (PMC11641727; doi:10.3390/ijms252312497)
Supplement: Supplementary file 1 [file ijms-25-12497-s001.zip › Table S2.pdf]

Table S2 *E. coli* CPS Screening and JSSK01 phage host range

Bacterial strains	*kpsMTII* gene	*waaL* gene	JSSK01
27356	-	[c]N/D	-
28314	[a]K1-related	[d]R1	-
42544	-	N/D	-
17331	-	N/D	-
19837	K1-related	R1	-
50076	-	N/D	-
55333	K1-related	N/D	-
57271	-	N/D	-
53996	-	N/D	-
60310	-	N/D	-
73266	-	N/D	-
71464	-	-	-
70751	[b]K1	R1	+
65501	-	N/D	-
69596	-	N/D	-

26165	-	N/D	-
80307	-	N/D	-
78943	-	N/D	-
78030	K1	R1	+
135545	-	R1	-
18h1k	K1	-	+
94852	K1-related	-	-
105378	-	N/D	-
107173	-	N/D	-
113353	K1-related	N/D	-
124505	K1-related	N/D	-
BCRC10675	O1:K1:H7	N/D	+
CRE14	-	N/D	-
CRE40	-	N/D	-
G0001	-	N/D	-
G0002	K1	R1	+
G0003	-	N/D	-

G0005	-	-	-
G0012	K1-related	-	-
G0030	K1-related	-	-
G0032	K1	R1	+
G0035	K1-related	R1	-
G0047	K1-related	N/D	-
G0048	K1-related	N/D	-
G0057	K1-related	R1	-
G0063	K1-related	-	-
G0072	K1-related	N/D	-
G0077	K1	R1	+
G0084	-	N/D	-
G0090	K1	R1	+

G0096	-	R1	-
G0111	K1	R1	+
G0112	K1-related	N/D	-
G0116	K1-related	N/D	-
G0117	K1	R1	+
G0120	K1-related	-	-
G0121	K1-related	N/D	-
G0128	K1-related	N/D	-
G0134	K1-related	N/D	-
G0144	K1-related	N/D	-
G0145	-	N/D	-
G0148	-	N/D	-
G0150	-	N/D	-
G0159	K1-	R1	-

	related		
G0170	K1-related	N/D	-
G0174	K1-related	N/D	-
G0182	K1	R1	+
G0184	K1-related	N/D	-
G0190	K1	R1	+
G0196	-	N/D	-
G0205	K1-related	N/D	-
G0209	K1	R1	+
G0215	-	N/D	-
G0218	K1	R1	+
G0220	-	N/D	-
G0222	-	R1	-
G0229	-	N/D	-
G0232	K1	R1	+
G0237	K1-	N/D	-

	related		
G0239	K1-related	N/D	-
G0243	K1	R1	+
G0255	K1	R1	+
G0259	-	N/D	-
G0273	K1-related	N/D	-
G0277	K1-related	-	-
G0283	-	N/D	-
G0284	K1-related	N/D	-
G0316	-	N/D	-

[a]K1-related: presence of the *kpsMTII* gene and absence of the K1 CPS; [b]K1: presence of the *kpsMTII* gene and presence of the K1 CPS; [c]N/D: Not determined; [d]R1: presence of the *waaL* gene and presence of R1 LPS; +: phage sensitivity; -: not sensitive/no particular target
