# Supplementary material for: Therapeutic and Diagnostic Potential of a Novel K1 Capsule Dependent Phage, JSSK01, and Its Depolymerase in Multidrug-Resistant Escherichia coli Infections
Source: Int J Mol Sci. 2024 Nov 21;25(23):12497. doi: 10.3390/ijms252312497 (PMC11641727; doi:10.3390/ijms252312497)
Supplement: Supplementary file 1 [file ijms-25-12497-s001.zip › Table S3.pdf]

1. Molecular cloning of endo-Nacetylneuraminidase (*orf40*) gene

To clone *orf40* (endo-N-acetylneuraminidase), the sequence was amplified from purified phage DNA using primers with *BamH*I and *Xho*I restriction sites (5´-GGA TCC ATG TCA AGC GGA TGC GG-3´ and 5´-CTC GAG TTA TTT ATC TTC TAG TGC TGC CAG C-3´). PCR was performed with PFU DNA polymerase under the following conditions: initial denaturation at 94°C for 10 min, 30 cycles of 94°C for 1 min, 54°C for 45 sec, 72°C for 3 min, and a final extension at 72°C for 10 min. The product was visualized via 0.8% agarose gel electrophoresis and purified using a PCR clean-up kit.

Ligation was carried out with T4 DNA ligase, combining the purified PCR product and the pGEM-T Easy vector at a 10:1 ratio, incubated at 4°C for 16–18 hours. The ligation mix was heat-shocked into *E. coli* DH5α competent cells, and transformed clones were identified via blue/white screening, followed by colony PCR to confirm the presence of *orf40*.

The confirmed plasmid, pGEM-T Easy_*orf40*-04, was subjected to restriction digestion with *Pml*I, *Xho*I, and *BamH*I, and fragment sizes were analyzed on a 0.8% agarose gel. To subclone *orf40* into the pET30a(+) expression vector, both pET30a(+) and pGEM-T Easy_*orf40*-04 were double-digested with *BamH*I and *Xho*I, purified, and ligated. The resulting construct was transformed into BL21(DE3) competent cells and plated on LB/Km plates.

Transformants were screened, and plasmid verification by restriction digestion confirmed the successful cloning of *orf40* in the pET30a(+) vector, with the pET30a(+)_*orf40*-01 clone being identified as positive.

1.1Confirmation of transformants by Quick screening and Colony PCR

The obtained colony was mixed within 100 µl of Quick screening buffer (50 mM Tris, 3% SDS, pH12.5) and incubated at a 65 °C water bath for 10 min. Add the equal volume of phenol & chloroform in a (1:1) ratio, and ensure the mix by inverting 50 times. Centrifuge the phenol-chloroform added sample at 12k rpm for 5 min. Transferred the supernatant and verified the presence of the plasmid in 0.7% agarose gel. All the transformant colonies are screened by colony PCR.
