# Supplementary figures and images for: Therapeutic and Diagnostic Potential of a Novel K1 Capsule Dependent Phage, JSSK01, and Its Depolymerase in Multidrug-Resistant Escherichia coli Infections
Source: Int J Mol Sci. 2024 Nov 21;25(23):12497. doi: 10.3390/ijms252312497 (PMC11641727; doi:10.3390/ijms252312497)

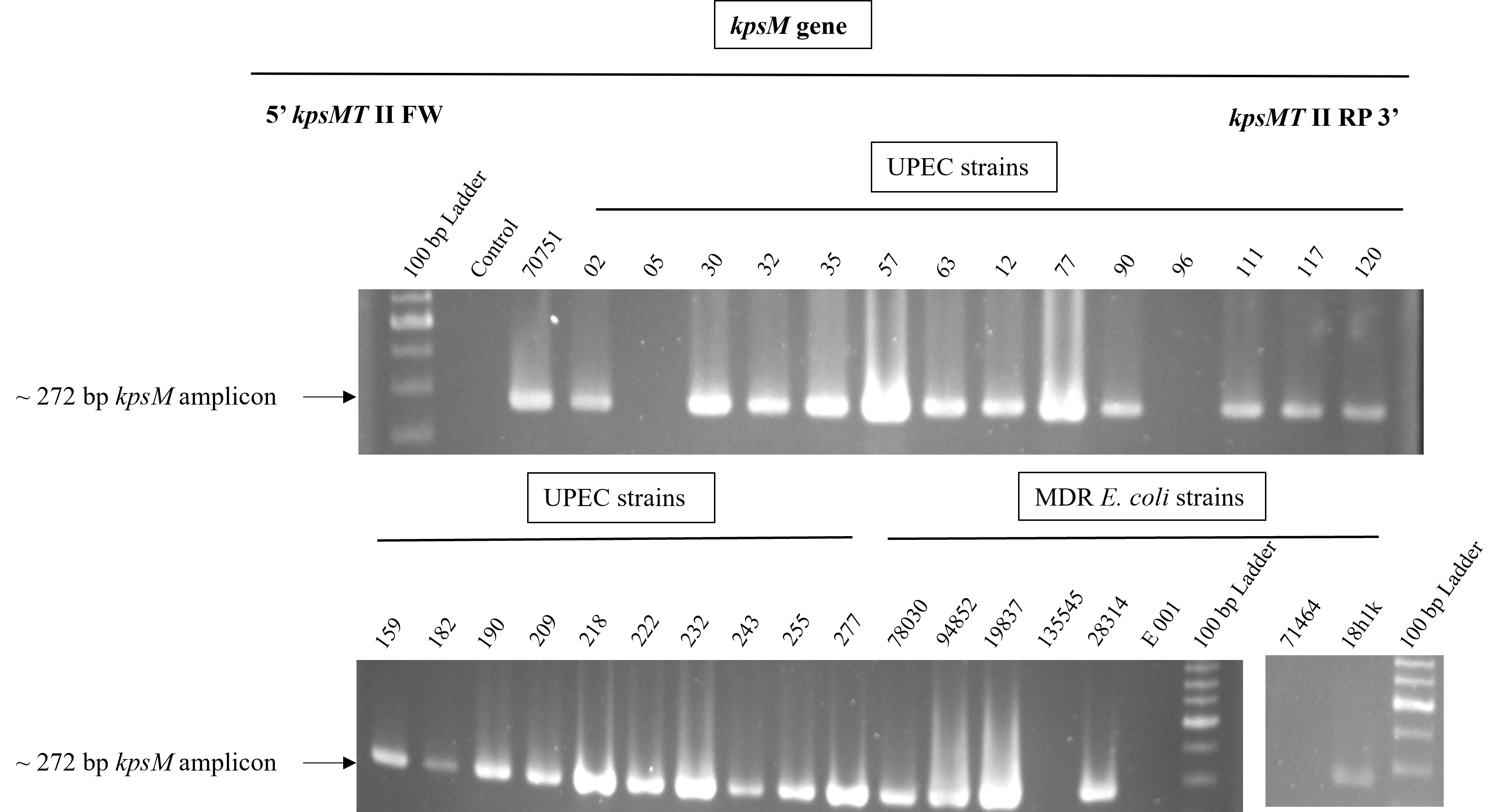

Supplement: Supplementary file 1 [file ijms-25-12497-s001.zip › Fig S1.tif]

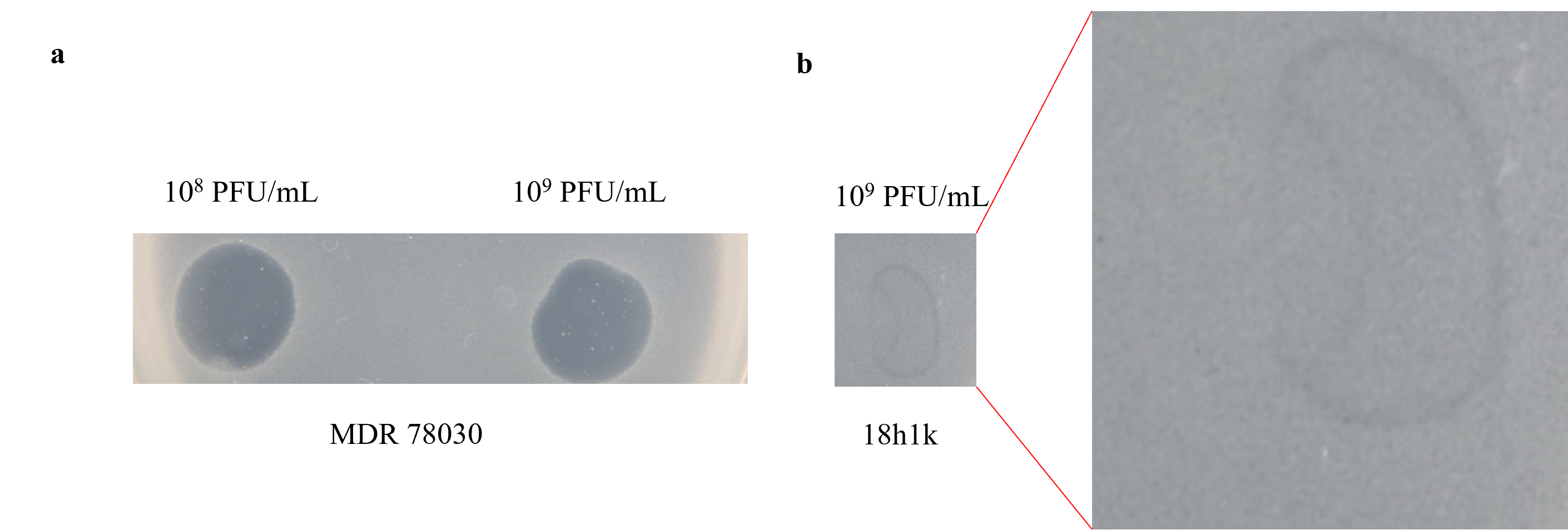

Supplement: Supplementary file 1 [file ijms-25-12497-s001.zip › Fig S2.tif]

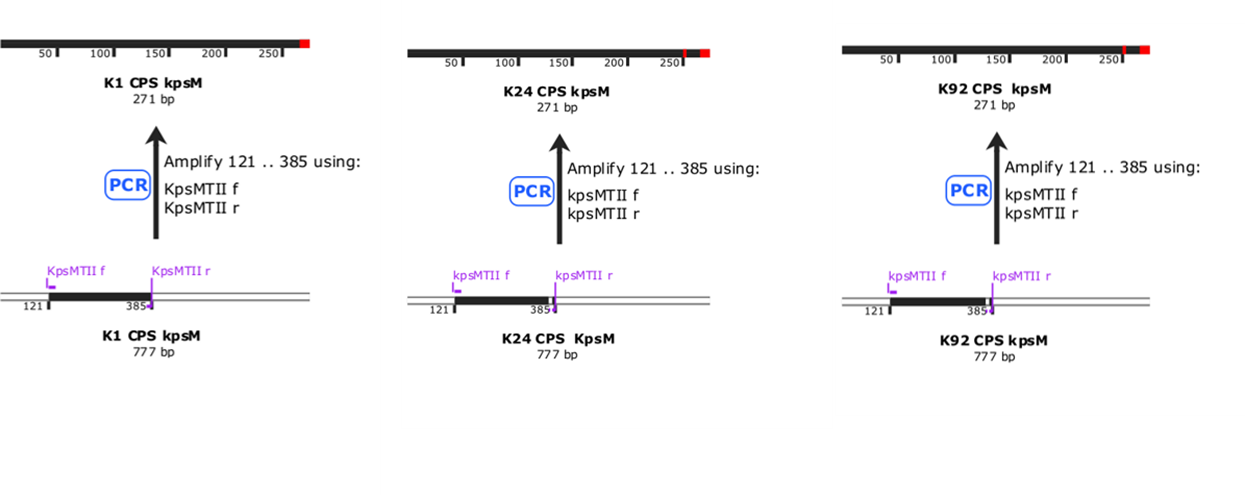

Supplement: Supplementary file 1 [file ijms-25-12497-s001.zip › Fig S3.tif]
